# Timely Questions Emerging in Chronobiology: The Circadian Clock Keeps on Ticking

**DOI:** 10.5334/jcr.237

**Published:** 2024-04-02

**Authors:** Sangeeta Chawla, John O’Neill, Marina I. Knight, Yuqing He, Lei Wang, Erik Maronde, Sergio Gil Rodríguez, Gerben van Ooijen, Eduardo Garbarino-Pico, Eva Wolf, Ouria Dkhissi-Benyahya, Anjoom Nikhat, Shaon Chakrabarti, Shawn D. Youngstedt, Natalie Zi-Ching Mak, Ignacio Provencio, Henrik Oster, Namni Goel, Mario Caba, Maria Oosthuizen, Giles E. Duffield, Christopher Chabot, Seth J. Davis

**Affiliations:** 1 Department of Biology, University of York, York YO105DD, UK; 2 MRC Laboratory of Molecular Biology Cambridge, UK; 3 Department of Mathematics, University of York, York YO105DD, UK; 4 Key Laboratory of Plant Molecular Physiology, CAS Center for Excellence in Molecular Plant Sciences, China National Botanical Garden, Beijing 100093, CN; 5 Institut für Anatomie II, Dr. Senckenbergische Anatomie, Goethe-Universität Frankfurt, Theodor-Stern-Kai-7, 60590 Frankfurt, DE; 6 School of Biological Sciences, University of Edinburgh, Max Born Crescent, Edinburgh EH9 3BF, UK; 7 Universidad Nacional de Córdoba (UNC), Facultad de Ciencias Químicas, Departamento de Química Biológica Ranwel Caputto, Córdoba, AR; 8 CONICET-UNC, Centro de Investigaciones en Química Biológica de Córdoba (CIQUIBIC), Córdoba, AR; 9 Institute of Molecular Physiology (IMP), Johannes Gutenberg-University Mainz, Hanns-Dieter-Hüsch- Weg 17, 55128 Mainz, DE; 10 Inserm, Stem Cell and Brain Research Institute U1208, Univ Lyon, UniversitéClaude Bernard Lyon 1, 18 Avenue du Doyen Lépine, 69500, Bron, FR; 11 Simons Centre for the Study of Living Machines, National Centre for Biological Sciences, Bangalore, Karnataka 560065, IN; 12 Edson College of Nursing and Health Innovation, Arizona State University, Phoenix, AZ, US; 13 Department of Medicine, University of Arizona, Tucson, AZ, US; 14 Department of Biology and Department of Ophthalmology, University of Virginia, Charlottesville, VA, US; 15 Institute of Neurobiology, Center for Brain, Behavior & Metabolism (CBBM), University of Luebeck, 23562 Luebeck, DE; 16 Biological Rhythms Research Laboratory, Department of Psychiatry and Behavioral Sciences, Rush University Medical Center, Chicago, IL, US; 17 Centro de Investigaciones Biomédicas, Universidad Veracruzana, Xalapa, Ver., MX; 18 Department of Zoology and Entomology, University of Pretoria, Pretoria, ZA; 19 Mammal Research Institute, University of Pretoria, Hatfield, ZA; 20 Department of Biological Sciences, Galvin Life Science Center, University of Notre Dame, Notre Dame, US; 21 Department of Biological Sciences, Plymouth State University, Plymouth, NH 03264, US; 22 State Key Laboratory of Crop Stress Biology, School of Life Sciences, Henan University, Kaifeng 475004, CN

**Keywords:** Circadian, Redox, Modeling, Photoperiod, Allelic variation, phenotypes, Algae, Liquid-liquid phase separation, Structural biology, Retina, Melatonin, Synchrony, Entrainment, Immunity, Sleep, Food-entrainable oscillator, Chronotherapy, Circadian alignment, Urbanisation, Crop plants, Circatidal, Circalunar, Mosquito biting, Seasonal migration, Imaging

## Abstract

Chronobiology investigations have revealed much about cellular and physiological clockworks but we are far from having a complete mechanistic understanding of the physiological and ecological implications. Here we present some unresolved questions in circadian biology research as posed by the editorial staff and guest contributors to the Journal of Circadian Rhythms. This collection of ideas is not meant to be comprehensive but does reveal the breadth of our observations on emerging trends in chronobiology and circadian biology. It is amazing what could be achieved with various expected innovations in technologies, techniques, and mathematical tools that are being developed. We fully expect strengthening mechanistic work will be linked to health care and environmental understandings of circadian function. Now that most clock genes are known, linking these to physiological, metabolic, and developmental traits requires investigations from the single molecule to the terrestrial ecological scales. Real answers are expected for these questions over the next decade. Where are the circadian clocks at a cellular level? How are clocks coupled cellularly to generate organism level outcomes? How do communities of circadian organisms rhythmically interact with each other? In what way does the natural genetic variation in populations sculpt community behaviors? How will methods development for circadian research be used in disparate academic and commercial endeavors? These and other questions make it a very exciting time to be working as a chronobiologist.

## Introduction

Circadian rhythms have long been known to play a fundamental role in the regulation of diverse physiological processes across a wide range of organisms. Recent advances in genetics, molecular biology, and imaging technologies have provided new insights into the mechanisms underlying circadian rhythms and their relationship to physiological and developmental outcomes. However, many questions remain unanswered, including the location and underlying mechanisms of “other clocks” that allow for synchronization to periodic environmental effects, the coupling of cell-cell communication in circadian rhythms, and the heterodimer relationship of transcription factor targets to move from a core clock to 5–30% of the transcriptome being rhythmic over the day. These unresolved questions, along with emerging methods and mathematical approaches to resolve them, underscore the continued relevance of circadian biology research. Furthermore, similarities between oscillators in various kingdoms suggest a fundamental relationship between structural and biochemical features, while the relation of genetics to physiological and developmental outcomes highlights the potential for circadian rhythms research to advance our understanding of complex biological systems.

Circadian oscillators are present in organisms ranging from bacteria to humans, indicating analogous conservation. Despite the differences in the structure and biochemistry of circadian oscillators across various kingdoms, there are remarkable similarities in the underlying mechanisms. For example, the negative feedback loop consisting of clock genes and their protein products is present in all known circadian oscillators. In addition, post-translational modifications, such as phosphorylation, play a crucial role in regulating the activity of clock proteins in diverse organisms. These similarities in molecular mechanisms suggest that the fundamental principles of circadian regulation have been conserved during evolution. Studies of the genetics of circadian rhythms have provided insights into how these mechanisms contribute to physiological and developmental outcomes. Disruptions in the circadian clock have been linked to a variety of disorders, including metabolic syndrome, cancer, and neuropsychiatric diseases. The circadian clock has clearly been a target for agricultural domestication as well. Thus, understanding the genetics of circadian rhythms can have significant implications for plant, animal, and human health.

New mathematical and imaging approaches have been instrumental in advancing our understanding of circadian rhythms. Mathematical models based on ordinary differential equations (ODEs) have provided insights into the complex interactions of genes and proteins that govern the circadian clock. These models have been refined and validated using imaging techniques, such as bioluminescence imaging and fluorescent microscopy, which allow researchers to visualize and quantify molecular events in real time. Furthermore, single-cell approaches have enabled researchers to probe the dynamics of the clock at a more detailed level, revealing previously hidden features of the clock’s behavior. New advances in imaging have led to breakthrough discoveries in circadian research. The spatiotemporal organization of proteins has challenged cellular imaging platforms, and engineering modifications have solved this for long-term 4D imaging of cells and organisms.

Genetics plays a crucial role in circadian rhythms research, as demonstrated by the discovery of clock genes in model organisms. These genes encode proteins that are integral to the functioning of the circadian clock, and mutations in these genes can lead to disruptions in the timing of physiological and developmental processes. By identifying and characterizing clock genes, researchers have been able to elucidate the biochemical pathways that underlie circadian rhythms, and to gain insights into how the clock interacts with other physiological systems. Moreover, genetic studies have revealed that the circadian clock has diverse roles in development, disease, and disease resistance. This has implications for fields ranging from crop breeding to chronotherapy to cancer research. It also has widespread implications to conservation biology in ecological contexts. Thus, genetics remains an essential component of circadian rhythms research, as it provides a foundation for understanding the mechanistic basis of these complex biological rhythms.

Over the next 5 to 10 years, experts predict that research in the field of circadian rhythms will continue to focus on unraveling the fundamental mechanisms underlying these oscillations. There will likely be an increased emphasis on understanding the interaction and coupling of different cell types and organs in the body, as well as the role of environmental cues in shaping circadian rhythms. New and innovative imaging and modeling techniques will also continue to drive progress in the field, allowing researchers to gain a more comprehensive understanding of the complex interplay between genes, proteins, and other molecules involved in the regulation of circadian rhythms. Induced mutagenesis to create clock allele combinations will be part of crop breeding as plants are “migrated” away from the equator in response to climate change. Additionally, there will likely be a growing focus on the potential clinical applications of circadian biology research, with the development of new therapies and interventions for a range of conditions associated with disrupted or irregular circadian rhythms.

Overall, and as highlighted by individual insights below, the next decade promises to be an exciting and transformative period for circadian rhythms research, with the potential to uncover new insights into the workings of the biological clock and its role in health and disease.

**Section: Redox, metabolism and cellular homeostasis**

John O’Neill

Circadian rhythms are vital for optimizing energy efficiency through the temporal organization of metabolic processes, including protein synthesis and autophagy. These rhythms rely on coordinated flux through metabolic pathways involving redox reactions, facilitated by enzymes such as GAPDH and their coupling with cofactor pools like NADH:NAD+ and NADPH:NADP+. The activity of respiratory electron transport chains and many oxidoreductases also show daily variation that impacts redox balance through the generation of reactive oxygen species (ROS) like superoxide and hydrogen peroxide (H_2_O_2_). Consequently, daily variations in redox cofactor balance occur across diverse eukaryotic contexts under different conditions. Physiological levels of H_2_O_2_ (<10 µM) function as signaling molecules, oxidizing redox-sensitive protein thiols, primarily low pKa cysteines, to stimulate disulfide formation and modulate protein activity. Cellular antioxidant systems, including glutathione (GSH) and peroxiredoxins (PRX), then reduce H_2_O_2_, which maintains redox homeostasis. Highly abundant and ubiquitous antioxidant PRX proteins exhibit circadian rhythms in their oxidation state that persist in the absence of rhythmic clock gene activity, in isolated red blood cells, for example [1,2]. Subsequent work revealed this was largely explained by rhythmic ROS production and degradation of over-oxidised forms of the protein (PRX-SO_2/3_), rather than an autonomous “redox oscillator” [3,4]. Acute oxidative stress certainly elicits circadian phase shifts, however, chronic redox stress has minimal impact on circadian period [5,6]. This suggests that daily redox variation is an output from, and can function as an input to, the cellular clockwork but is not essential to the timing mechanism. Understanding the causal connections between metabolism, related redox changes, protein homeostasis and circadian regulation is essential for comprehending daily regulation of cell physiology and diseases associated with disrupted circadian rhythms. In the next few years, it will be interesting to establish how much can be explained by daily rhythms in the activity of mechanistic target of rapamycin complexes (mTORC) [7].

**Section: Mathematics of circadian rhythms**

Marina Knight

The mathematics of circadian rhythms has a rich history, deeply rooted in their inherent stochastic nature as time series. However, recent advancements have unveiled the nonstationary nature of these rhythms and their susceptibility to feature missing or irregularly sampled data, which in turn poses significant challenges for their accurate analysis. Traditional statistical methods, commonly employed for analyzing time-dependent processes, often fail when confronted with the simultaneous presence of nonstationarity, irregular sampling [8], and replication [9], which are all prevalent characteristics of circadian data. This limitation leads to biased results and erroneous conclusions. Thus, there is a pressing need to develop a novel theoretical and methodological framework that effectively addresses the joint issues of nonstationarity, irregular sampling, and replication commonly encountered in circadian rhythm studies. To overcome these challenges, the article proposes integrating second-generation wavelet techniques, also known as lifting schemes, with a locally stationary modeling framework [10,11]. Although developing such a framework is not straightforward, recent progress in the field of time series analysis, particularly in the context of circadian data, offers promising avenues for advancement [12,13]. Such a combined approach holds the potential to achieve a more precise, unbiased, and robust analysis of circadian data and to enhance our understanding of the multiscale nature of circadian rhythms and their implications for various aspects of health and disease. The proposed modeling framework would furthermore enable the prediction and quantification of the stochastic variations induced by environmental stressors such as pollution or climate change. Ultimately, this advancement can improve our ability to address circadian-related issues and inform strategies for mitigating the effects of environmental challenges on circadian rhythms.

**Section: Rice clock**

Yuqing He, Lei Wang

The circadian system in higher plants, particularly in angiosperms, has been extensively studied in the model plant Arabidopsis, revealing the crucial roles of core clock components in crop plants [14,15,16,17]. These components influence processes such as latitudinal adaptation, growth and development, and environmental stress tolerance. Clock genes in crops have undergone domestication and remain valuable gene pools for breeders. However, to advance our understanding of the circadian clock in crops and harness this knowledge for molecular design breeding and green agriculture, several key questions need to be explored. Firstly, it is important to investigate whether there are novel or specific clock components in crops. Crop-specific regulators, such as OsGhd7 in rice and GmE1 in soybean, act as key components in regulating photoperiodic sensitivity networks. Identifying crop-specific clock components that interact with light signals to determine photoperiod sensitivity would be highly valuable. Secondly, the specific roles of clock homologs in crops need systematic elucidation. Crops like wheat, which are polyploid, possess multiple homologs of clock components compared to Arabidopsis. For example, even diploid rice has two distinct ELF3 homologs. While OsELF3.1 predominantly regulates heading date and salt tolerance, OsELF3.2 primarily modulates plant immunity through interaction with E3 ligase APIP6e [18,19,20]. Differentiating the redundant and specific roles of individual homologs in crops would provide deep insights. Thirdly, assembling the circadian system in crops comprehensively and meticulously is crucial. Given that crops likely have specific clock components and multiple homologs of core clock components, their circadian system may exhibit greater complexity than Arabidopsis. Additionally, the regulatory network of clock outputs could be more intricate in crops. Resolving these questions would provide a comprehensive understanding of how crop circadian clocks facilitate adaptation to daily and seasonal environmental changes and enable domestication across various latitudes. This knowledge would greatly contribute to regional adaptation breeding for higher crop yields and enhanced tolerance to biotic and abiotic stresses. Fully investigating the circadian clock in crops has significant implications for agriculture. Understanding the specific clock components and their roles could lead to more efficient molecular design breeding and the development of green agriculture techniques. By systematically exploring and distinguishing the redundant and specific roles of individual homologs, researchers may identify genetic modification targets that improve crop yields and resilience. Overall, a comprehensive understanding of the circadian clock in crops has the potential to significantly impact global food security and the agricultural industry.

**Section: Allelic variation in plant clock genes: why?**

Seth J. Davis

The circadian clock of plants holds promise to adapt ecological and agricultural plants to the consequences of climate change. Numerous studies in wild annuals have revealed the earlier flowering in response to early warming in the Spring. Extensive allelic variation exists in clock genes and this has been detected in natural clines in several plants in differing climatic contexts. Similar consequences in crops are being alleviated by growing our major crops to every increasing migration to the poles, and this has negative effects on yield given altered photoperiodic reactions. Moving forward the agricultural and ecological communities will undoubtably assess haplotype diversity at major clock genes and photoperiodic input factors. To foster food security in response to our current 8 billion people, with another 2 billion expected over the next 25 years, exploiting this natural allelic variation detected in crops will be expanded by novel genetic and genomic approaches to create large germplasms at clock genes to facilitate this latitudinal migration that is ongoing. From the research on the circadian clock of plants, a better understanding of the molecular mechanisms underlying plant responses to environmental cues such as temperature and photoperiod can be gained. This knowledge can be utilized to adapt crops to the challenges of climate change, which is expected to have significant impacts on global food security. By identifying the allelic variation in clock genes and photoperiodic input factors, plant breeders can develop crop varieties that are better suited to changing environmental conditions. Furthermore, advances in genetic and genomic technologies will allow for the creation of large germplasms at clock genes, which can be used to increase crop yield and facilitate the ongoing migration of crops. By exploiting natural allelic variation in clock genes and using advanced genetic and genomic tools, plant breeders will create crops that are more resilient to environmental stressors and pathogens, and have higher yields. This can help to ensure food security for the growing human population, while minimizing the negative impacts of climate change on agriculture.

**Section: Clock gene knockout phenotypes not directly connected to circadian rhythms in mice**

Erik Maronde

My most pressing question in the chronobiological field is how the deletion of e.g. Period1 in mice translates into complex phenotypes of the Musculo-skeletal system, whole body-size and behavior [21,22], especially since deletion of period1 does not lead to arrhythmicity under constant conditions [23]. In other words, we would like to find out which gene-, RNA- and Protein- networks are affected by the period1-deficiency that are phenotypically expressed in different structures of some bones, but no others, change some behaviors, but not seemingly related ones. We would also like to find out if there is a hierarchical order of compensation and non-compensation that mounts into the whole-body differences in period1-deficient mice in comparison to their wild-type and heterozygotic siblings. To understand the impact of Period1 deletion in mice, researchers need to perform extensive studies to identify the underlying gene, RNA, and protein networks affected by the deficiency. These networks may be responsible for the complex phenotypes observed in the musculo-skeletal system, whole body size, and behavior of the mice. By exploring these networks, researchers may identify potential therapeutic targets to treat disorders related to the musculo-skeletal system, behavior, and other physiological processes in humans. Additionally, understanding the hierarchical order of compensation and non-compensation that leads to the whole-body differences in period1-deficient mice may provide insights into the mechanisms underlying circadian regulation of physiological processes. This information may be useful for developing treatments for various disorders linked to circadian rhythm disruptions, such as sleep-, mood-, and metabolic disorders. It may also pave the way for the development of new genetic and genomic approaches to treat various diseases linked from the circadian clock to developmental outcomes, which would have a significant impact on public health.

**Section: Algal clocks**

Sergio Gil Rodríguez and Gerben van Ooijen

Algae are a morphologically and taxonomically diverse group of photosynthetic organisms, ranging from prokaryotes as cyanobacteria to eukaryotes as seaweeds, and are globally widespread from oceans to soils. Circadian research in eukaryotic species of algae has mostly been limited to members of the green algae (chlorophytes), which provided model systems of reduced complexity [24,25]. Early examples include studies in the giant cells (~10 cm) of *Acetabularia crenulata* that identified cellular circadian rhythms without a nucleus [26,27]. These experiments were mirrored in one of the smallest species of algae (~1 µm; *Ostreococcus tauri*) by the identification of non-transcriptional rhythms that were found to be conserved across all eukaryotes [28,29]. The fundamental outstanding questions from this line of algal circadian research must be: how are non-transcriptional rhythms generated and maintained? To what extent do they couple to and influence the TTFL-based timekeeping system in the nucleus? However, circadian rhythms in algae have wider importance than by providing model cells. Algal communities represent 10 times more biomass than all descendant land plants [30] and contribute to half of the global oxygen production and CO_2_ fixation [31]. In contrast to green algae, we know little about circadian rhythms in ecologically critical groups such as red algae (rhodophytes), brown algae (phaeophytes), diatoms (bacillariophytes) or dinoflagellates (pyrrhophytes) [25]. Characterization of the molecular clocks from the diverse taxonomy of algae would provide much-needed answers about the evolution of timekeeping and could also provide a step-change in the biotechnological applications of algae to produce food, feed, high-value compounds, and biofuels. The impact of studying circadian rhythms in algae could be significant in various fields. Algal communities contribute significantly to global atmospheric chemistry, and understanding the molecular clocks of different algae species can provide insights into the evolution of timekeeping. Additionally, the research could lead to biotechnological advancements in producing food, high-value compounds, and biofuels. However, the pressing questions that need to be addressed are the functional relevance of rhythmicity for algae and how widespread biological rhythms are in algae. Answering these questions can help us understand the phenotypic plasticity of algae under changing environmental conditions and the vital role they play as lungs of the Earth.

**Section: Liquid-Liquid Phase Separations and Intracellular RNA-Protein Condensates: potential interactions with the Circadian System**

Eduardo Garbarino-Pico

Over the past two decades, researchers have identified and characterized a novel class of organelles known as membraneless organelles or RNA granules [32]. These organelles lack membrane boundaries but exhibit selective enrichment of specific molecules while excluding others. They arise through a process called liquid-liquid phase separation (LLPS) triggered by the accumulation of biopolymers like RNA and proteins. The multivalent nature of these molecules enables the rearrangement of interactions necessary for their formation. Despite extensive research, the involvement of biocondensates in circadian rhythm regulation and their interaction with biological clocks remain largely unknown.

Some studies have reported rhythmic behavior in specific biocondensates such as processing bodies (PBs) and stress granules (SGs). PBs, found in the cytoplasm, undergo changes in size and abundance in response to stress, and their presence shows rhythmicity in different cell types [33,34]. These compartments contain silenced and deadenylated transcripts, mRNA decay factors, and RNA-binding proteins. The precise function of PBs is still subject to debate, as some studies suggest a reservoir-like role for PBs, allowing certain mRNAs to exit and reassemble polysomes for translation, while others demonstrate mRNA degradation within these compartments [35,36,37]. The daily oscillations of PBs may explain discrepancies between circadian profiles of mRNAs and their corresponding proteins. Rhythmic transcripts can encode proteins with invariant levels or exhibit different phase relationships with mRNA oscillations. The localization of mRNA to PBs prevents protein translation, and changes in mRNA composition over time may contribute to the temporal regulation of transcript silencing and degradation. Another example of rhythmic biocondensates is stress granules (SGs), which show daily fluctuations in formation, particularly in response to oxidative stress [38]. The rhythmic phosphorylation of the translation initiation factor eIF2α is associated with SG formation and has been observed in various organisms, including the mouse suprachiasmatic nucleus [39] and the fungus Neurospora crassa [40]. These changes in phosphorylation are linked to the circadian translation of certain mRNAs, some of which encode components of cytoplasmic mRNA ribonucleoprotein granules similar to PBs and SGs. Analyzing these granules at opposing circadian phases reveals differences in their numbers, indicating a regulatory influence of the circadian system [41]. Various factors governing biocondensate formation exhibit circadian oscillations, such as daily changes in RNA and protein content, rhythmic levels of specific molecules, fluctuations in ion concentrations, and other metabolites [42,43,44]. Given the involvement of biocondensates in essential cellular processes like chromatin state, transcription, splicing, transport, storage, decay, and translation of transcripts, it is reasonable to predict the existence of bidirectional regulatory pathways between biological clocks and different types of biocondensates in the future. Further exploration of these regulatory mechanisms will contribute to a deeper understanding of the circadian regulation of biocondensates and its implications in cellular function.

**Section: Structures and clocks**

Eva Wolf

Structural biology has provided significant insights into the cyanobacterial clock by elucidating the phosphorylation timer of the KaiABC complex [45,46]. For the mammalian circadian clock, 3D-structures and biophysical descriptions of the PER, CRY, BMAL1, CLOCK and CK1 core components and of CRY/PER2-, CRY2/FBXL3- and CLOCK/BMAL1 complexes are available [46,47]. Furthermore, single particle electron microscopy has provided first insights into the architecture of cytosolic and nuclear clock protein complexes, including the multi-subunit early repressive CRY/PER containing complex [48]. An important next step will be to structurally characterize the daily changing interactions of transcriptional co-repressors or co-activators. These include chromatin modifiers, with CLOCK/BMAL1, PER or CRY, at a higher resolution. Such structures will provide molecular mechanistic insights into the assembly, disassembly and interconversion of active and repressive complexes and elucidate associated competitive or cooperative binding events. In this context, the interactions of CRY1 and CRY2 as well as of PER1, PER2 and PER3 should be individually analyzed to elucidate the non-redundant roles of these homologues in the circadian oscillator. Additionally, structural insights into posttranslational modifications (PTMs) of clock proteins, such as phosphorylation, ubiquitylation and acetylation are essential to fully understand circadian timing [49,50]. While PTMs of eukaryotic clock proteins are often located in disordered protein regions and therefore not easily accessible to high resolution structural analyses, structures of adjacent clock protein domains can still provide valuable insights into their relative locations in space [46,49]. Apart from the CRY2-FBLX3 complex structure [51], structural information for ubiquitin-dependent proteasomal degradation of clock proteins is still lacking. Further structures and biophysical studies of E3-ligases in complex with their clock protein targets (e.g. PER-SLIMB in Drosophila, PER1/2-TRCP in mammals, Frq-FWD-1 in Neurospora, ZTL-TOC/PRR5 in plants) would provide important mechanistic insights into circadian timing via clock protein degradation. Atomic-level 3D-structures have been and will be instrumental for the development of small molecules targeting clock proteins to interfere with circadian function [52] and are key to understand and improve the isoform selectivity of such compounds, for example for CRY1 vs CRY2 [53]. Furthermore, upcoming structures of clock protein complexes may open up new avenues for small molecule modulators targeting specific clock protein interactions, akin to the KL001 compound, that targets the CRY-FBXL3 interface to enhance CRY stability [51,53]. Structural studies of plant circadian clock proteins are so far largely limited to light receptors and their complexes with direct interactors, including the Cryptochromes and their complexes with the CIB transcription factor [54] and the BIC inhibitor [55], Phytochrome B [56] and the LKP2- Gigantea complex [57]. Apart from LUX ARRHYTHMO located in the evening complex of the plant circadian clock [58], no high resolution structures of plant core clock components are available. Such structures – while potentially challenging to get – are highly desirable to provide a better mechanistic understanding of how the plant circadian clock is regulated by daily changing protein interactions, chromatin modifications, temperature changes as well as proteasomal degradation or posttranslational modifications of plant clock proteins [59,60]. The impact of the structural biology studies mentioned in the passage will be significant in understanding the mechanisms of circadian rhythms in both bacteria and mammals, and potentially in plants. By providing detailed structural information on the interactions and modifications of clock proteins, these studies will help to elucidate the assembly and disassembly of active and repressive complexes, as well as the competitive or cooperative binding events associated with circadian timing. Overall, the impact of the structural biology studies discussed in the passage will be to further advance our understanding of the fundamental mechanisms underlying circadian rhythms in various organisms, with potential applications in medicine and agriculture.

**Section: Retinal clocks**

Ouria Dkhissi-Benyahya

A myriad of circadian rhythms has been described in the mammalian eye. Most of them, if not all, are regulated by the retinal clock [61,62]. The retinal clock plays a crucial function in adapting retinal physiology to the environmental light/dark cycle, by regulating rod outer segment disc shedding and phagocytosis by the retinal pigment epithelium, expression of immediate early genes and opsin genes in photoreceptors, and dopamine/melatonin synthesis [62,63,64]. Furthermore, the retinal clock controls cell survival and growth processes in the eye including the susceptibility of photoreceptors to degeneration from light damage, during aging or retinal diseases [65,66,67]. A detailed microarray study revealed that over 10% of genes (including clock genes) are expressed in a circadian manner in the eye [68] and our recent genome-wide transcriptome profiling studies in non-human primate have uncovered a wide array (up to 80% of protein-coding genes) of ubiquitous and tissue-specific genes under circadian control [69]. Thus, circadian rhythmicity pervasively regulates major cellular functions thereby coordinating physiology and ensuring homeostatic balance with the environment [61,70]. Indeed, most up-to-date studies indicate that disruption or misalignment of circadian rhythms caused by genetic or environmental factors increases the risk for various diseases, comprising metabolic, inflammatory, cardiovascular, cancer, and mental pathologies. Similarly, the widespread control of signaling, metabolism, and gene expression by the retinal clock suggests that its integrity is critical for maintaining normal rhythmic responses and retina homeostasis. The disruption of retinal clock organization or its entrainment by light, induced by circadian misalignment or retinal diseases, may have major impacts on eye function and health [61,70,71]. Although recent data enriched our knowledge about retinal clocks, several questions still need to be resolved. A major goal is to understand how retinal clocks control retina physiology, synaptic communication, and neural network activity in response to environmental or physiological changes. Establishment of the specific clock function in each retina cell types from different vertebrate species requires further investigation to determine how the myriad of retinal circadian rhythms is controlled. Another question still raised is whether these cellular/layer clocks interact? Is there a major pacemaker driving slave oscillators? Finally, another important challenge will be to identify the photoreceptors, the signaling pathways, and the diffusible signals involved in the coupling and the light response of the retinal clock, a key step to understand how the retina adapt to the changing ambient light over a day. The impact of the circadian rhythms in the mammalian eye is pervasive, as it regulates major cellular functions and coordinates physiology to ensure homeostatic balance with the environment. Disruption or misalignment of circadian rhythms caused by genetic or environmental factors increases the risk for various diseases, including metabolic, inflammatory, cardiovascular, cancer, and mental pathologies. It is expected that research on ocular rhythms will lead to improvements in our visual health as well as improve our understanding of the cellular components of vision.

**Section: Dim light – Melatonin**

Anjoom Nikhat and Shaon Chakrabarti

Exciting research in the mid and late 20^th^ century fundamentally changed the way we think about circadian rhythms in organisms – from the centuries old idea that environmental cycles drive these rhythms, to a new and quantitative understanding that the rhythms are endogenous and simply entrained by the cycles of day and night, similar to coupled oscillators. These ideas have now paved the way for an exciting future in circadian biology, where quantifying inter-individual variations in the phase difference between body clocks and the environment (chronotypes) could potentially be leveraged to personalize therapy timing in different diseases [72]. This endeavor has received a tremendous boost from recent advances in sequencing, imaging and computational methods, which are now allowing measurements of gene expression in a high throughput manner with single cell and spatial resolution [73,74]. These new-age technologies may therefore allow inferring body or even tissue-time of individuals from single time-point measurements, thus circumventing the need for elaborate and time-consuming temporal measurements of the more standard DLMO (dim-light-melatonin-onset) [75,76,77,78,79]. While some advances in these areas have already been made, there remains enormous scope for improving upon the accuracy of single time point measurements as well as predictions of time across tissue types. Furthermore, whether there exists fundamental limits to time-telling at single cell resolution remains unclear, where noisy gene expression may preclude accurate inference of circadian phase [80]. Solving these challenges along with developing minimally invasive and clinically implementable time inference protocols for individual patients remain the next big frontiers in chronotherapy research [76,81,82]. The ability to infer spatially resolved circadian phase in tissues, if possible, will also lead to a deeper understanding of why circadian clocks often become dysfunctional in diseases such as cancer. The impact of this research is that it can revolutionize our understanding of circadian rhythms in real-world settings. It can pave the way for future personalized therapy timing in different diseases. Challenges remain in developing minimally invasive and clinically implementable time inference protocols for individual patients and understanding the limits of time-telling at single-cell resolution as it relates to organismal physiological outcomes.

**Section: Circadian alignment in humans**

Shawn D. Youngstedt and Natalie Zi-Ching Mak

We believe crucial current important topics for the study of circadian rhythms in humans include the following. How does one define circadian misalignment? Abundant evidence suggests that simple, practical, and relatively inexpensive circadian measures derived from wrist actigraphy are associated with risks of cancer [83] and dementia [84], and with disease progression. Further confirmation is needed that inferences about the circadian system can be gleaned from actigraphic data (e.g., via correlation with the 24 rhythm of melatonin excretion), and what metrics of actigraphic data (e.g., level of amplitude) are indicative of poor or robust circadian synchronization. Determining this will allow the field to address the prevalence of circadian misalignment and its association with various health outcomes in the general population. Perhaps the most important current topic is whether improved synchronization of the circadian system (with bright light [85] or perhaps with multiple zeitgebers [86]) can reduce morbidity, and possibly increase longevity, in various patient populations, including individuals with cancer, Alzheimer’s Disease, and other maladies.

To address these important topics in circadian rhythm research, several areas of study and research should be performed. Firstly, more studies are needed to confirm that simple, practical, and relatively inexpensive circadian measures derived from wrist actigraphy are reliable indicators of circadian misalignment and synchronization. This will help the field to better understand the prevalence of circadian misalignment in the general population and its association with various health outcomes. Additionally, research should focus on developing effective interventions to improve circadian synchronization, such as bright light therapy or multiple zeitgebers. This research could potentially have a significant impact on the health outcomes of patient populations. Furthermore, there is a need for research to determine the impact of circadian misalignment on specific health outcomes and diseases. This could provide insight into the underlying mechanisms of these diseases and inform the development of targeted therapies.

**Section: Entrainment and human immunity**

Ignacio Provencio

Connections between the circadian and immune systems are emerging. Understanding how they interact will lead to a greater understanding of the mechanisms underlying pathologies resulting from circadian dysregulation. Almost two decades ago it was shown that a model of chronic jet-lag increases mortality in aged mice [87]. Chronic circadian disruption leading to a dysregulation of inflammatory responses is the likely culprit [88]. Exposure to inappropriately timed *Zeitgebers*, such as light and food, elicits or exaggerates inflammatory responses [89]. In fact, the salubrious effects of a calorie restricted diet are diminished if feeding occurs at an inappropriate circadian time [90]. Aging is associated with an increase in the expression of genes encoding inflammatory factors. A calorie restricted diet appropriately phased to the circadian cycle ameliorates these age-related changes in immune function.

These results suggest that circadian interventions may be effective tools to combat the ravages of aging. A better understanding of the connections between the circadian axis and the immune system will allow the development of such therapeutic strategies. Specifically, studies are needed to investigate the effects of inappropriately timed *Zeitgebers*, such as light and food, on the immune system and to determine the metrics for optimal circadian synchronization. Additionally, more research is needed to understand how calorie-restricted diets appropriately phased to the circadian cycle can ameliorate age-related changes in immune function. Such research could have significant impacts on the development of targeted therapeutic interventions for a variety of diseases and conditions associated with circadian dysregulation and inflammation.

**Section: How important is the clock for medicine?**

Henrik Oster

If I think back to the turn of the century, I remember the search going on around this time for identifying equivalents of fly clock genes in mammals. This was before fully sequenced genomes and before high-throughput sequencing. I particularly remember a few dead roads like the timeless story or fos as a regulator of light responses. These scientific questions of the past are relevant to our future. Now we are left with pressing questions for the next years in my humble opinion: how important is the clock for medicine? Which molecular rhythms are clock- which are non-clock coordinated? What really defines circadian disruption, and why does this matter? The search for mammalian equivalents of fly clock genes was a crucial endeavor that paved the way for modern chronobiology. Advances in molecular biology and genomics have led to the discovery of key players in the mammalian circadian system, including the CLOCK and BMAL1 transcription factors, which form a transcriptional activator complex that drives expression of clock genes. These genes, in turn, modulate the activity of the CLOCK-BMAL1 complex in a feedback loop that drives circadian rhythms in behavior, physiology, and gene expression. The importance of the clock for medicine has become increasingly clear in recent years. Studies have shown that circadian disruption, such as shift work or chronic jet lag, is associated with a wide range of adverse health outcomes, including cancer, metabolic disorders, and neurodegenerative diseases. Furthermore, the circadian system has been implicated in the timing and efficacy of drug treatments, with many drugs showing time-of-day dependent effects. A pressing question for the next years is to determine which molecular rhythms are truly clock-controlled and which are not. It is well established that many genes and physiological processes exhibit diurnal rhythms, but not all of them are under direct control of the core clock machinery. Understanding the scope and regulation of clock-controlled genes and processes will be important for developing strategies to combat circadian disruption and its associated health risks. Another important area of research is to define what truly constitutes circadian disruption and why it matters. For example, what is the threshold of light exposure or meal timing that leads to circadian misalignment? How does chronic circadian disruption affect physiological and cognitive function over time? Answering these questions will be critical for developing evidence-based recommendations for promoting healthy circadian rhythms in the general population. In summary, the search for clock genes in mammals was a crucial first step in modern chronobiology, but there is still much to learn about the regulation and impact of the circadian system in health and disease. Research on the importance of the clock for medicine, the scope of clock-controlled rhythms, and the definition and impact of circadian disruption will have far-reaching implications for public health and personalized medicine.

**Section: Circadian misalignment and sleep loss: fatigue and cognitive deficits and individual differences**

Namni Goel

It is well established that circadian misalignment and sleep loss produce fatigue and sleepiness, unstable wakefulness, deficits in cognitive functions, impaired mood affect regulation, and increased accidents and injuries. However, there are substantial individual differences (resilience and vulnerability) in the extent to which adults experience such deficits under these conditions; such differences are stable and trait-like and persist across months to years. These findings represent important phenotypic discovery steps toward harnessing and managing individual differences in resilience and vulnerability, but further research is needed. Furthermore, the mechanisms and predictive potential biomarkers underlying these individual differences remain elusive.

Understanding the mechanisms underlying these individual differences could provide valuable insights into the development of personalized interventions and therapies for sleep and circadian disorders. Experimental approaches will rely on novel measurement protocols and defined relationships to personal approaches to rhythmic health. One potential avenue of research is the examination of various “omics” markers, including genomics, metabolomics, proteomics, and transcriptomics. These markers could provide valuable information about the biological processes and pathways that contribute to individual differences in resilience and vulnerability to circadian misalignment and sleep loss. Another important area of research is the investigation of the long-term consequences of individual differences in resilience and vulnerability to circadian misalignment and sleep loss. For example, it is unclear whether individuals who are more resilient to sleep loss are also protected against the negative health consequences that can result from chronic sleep deprivation. Similarly, it is unclear whether interventions that improve resilience to circadian misalignment and sleep deprivation can also improve long-term health outcomes. Ultimately, understanding the mechanisms and biomarkers underlying individual differences in resilience and vulnerability to circadian misalignment and sleep deprivation could lead to the development of personalized interventions and therapies for sleep and circadian disorders. Such interventions could improve overall health and well-being and reduce the negative consequences associated with acute and chronic circadian misalignment and sleep loss.

**Section: Food entrainable mammalian oscillator**

Mario Caba

Future research on the food entrainable mammalian oscillator should focus on identifying the neural and molecular mechanisms that underlie this circadian system. Specifically, researchers should investigate the neuronal pathways and the gene expression patterns that are involved in food entrainment. Additionally, the functional properties and behavioral outputs of the food entrainable oscillator(s) need to be elucidated. This would involve examining the impact of food entrainment on physiological functions, including metabolism, hormone regulation, and sleep-wake cycles. Through this research, we can gain a better understanding of the interplay between circadian rhythms and feeding behaviors, which can have significant implications for human health. By understanding the molecular and neural mechanisms of the food entrainable oscillator, researchers may be able to develop therapies to treat circadian rhythm disorders, such as jet lag, shift work disorder, and certain sleep disorders. Additionally, understanding food entrainment could help in developing strategies to manage weight and metabolic disorders, as it is becoming increasingly evident that circadian rhythms play a significant role in metabolism and obesity. In summary, understanding the food entrainable mammalian oscillator is crucial for advancing our understanding of circadian rhythms, feeding behaviors, and overall health. Through continued research, we can uncover the underlying mechanisms of this system, which will ultimately lead to new treatments and interventions for a range of circadian rhythm disorders and metabolic diseases. How the clock links back to being hungry reveals a clear linking of what has been seen as a dichotomy between these.

**Section: Urbanization of clocks**

Maria Oosthuizen

Few people in southern Africa work in the field of circadian biology, and initial studies in the early 2000s were exclusively on African mole-rats. This has since been expanded to also include other rodent species. Currently the focus is on the effect of anthropogenic changes such as urbanisation, light at night and increasing temperatures on the circadian behaviour and physiology of rodents. Since activity patterns of animals have been shaped over many years according to their local habitats, temporal niche partitioning facilitates co-existence of species in terms of predation, resources and competition. Anthropogenic factors interfere with the environmental signals that animals use to entrain their activity, thus the responses of animals to these environmental changes can be used to predict their behavioural and physiological flexibility and survival. However Southern African rodents are overall rather understudied, therefore different species are firstly studied in the laboratory to obtain baseline information regarding their circadian systems, and thereafter measured in more natural settings with uncontrolled ambient temperature and light. Currently, the pressing question is how the circadian systems of wild caught rodents respond to anthropogenic changes in their environments. More broadly, research in the field of circadian biology in the southern hemisphere will connect ecological and seasonal changes in ways that have been limited to northern hemisphere-only research endeavors to date. The humanistic impact of anthropogenic changes on the circadian behavior and physiology of rodents in this region is virtually unstudied and requires urgent attention. It is not known how wild-caught rodents respond to anthropogenic changes in their environment and what adaptations or changes occur in their circadian rhythms, or to what extent laboratory based studies can be extrapolated to animals in their natural environments. To answer this question, we can use various techniques such as behavioral observations, activity monitoring, hormone assays, and gene expression analysis. By investigating the effect of anthropogenic changes on the circadian rhythms of rodents, researchers can gain insights into how these animals are adapting to changes in their environment and how these adaptations may affect their long-term survival. Additionally, this research could have important implications for understanding the impacts of human activity on wildlife, ecosystem health and biodiversity in southern Africa.

**Section: Temporal shifts in mosquito circadian biting rhythms**

Giles E. Duffield

The use of insecticidal-treated bed nets (ITBNs) and indoor residual spraying (IRS) has proven effective in protecting against malaria transmission by anopheline mosquitoes [91,92,93,94,95,96]. However, these interventions are exerting selective pressure on mosquito populations, leading to changes in their host-seeking behavior. Mosquitoes that traditionally bite humans during the night are now biting earlier or later in the night, and in some cases, even during the daytime [91,92,93,94,95,96]. These behavioral changes can occur within a few months of exposure and a few generations, suggesting a rapid adaptation process. It remains unclear whether these changes are caused by modifications in the mosquitoes’ circadian clock or alterations in the output processes that drive host-seeking behaviors. Mosquitoes possess diel and circadian rhythms, and a significant portion of their genomes is under rhythmic regulation [97,98,99]. Understanding the impact of long-term selective pressure on the circadian clock and its transcriptional-translational feedback loop (TTFL) components, known as clock genes [97,98,99], is crucial. Additionally, investigating the effects on the molecular and cellular factors involved in circadian and diel timing, such as transcriptional regulators, enzymes, neurotransmitters, receptors, and hormones, is essential. Moreover, man-made environmental changes, such as light pollution, are also influencing mosquito populations and their circadian clocks. Evidence suggests that artificial light at night can extend the duration of mosquito biting activity, particularly in the case of Aedes mosquitoes, which are day-active and have a preference for human hosts [100,101]. Urban environments, where these mosquitoes are more likely to encounter light pollution, may exacerbate the effects. Given these findings, research should focus on unraveling the mechanisms underlying the changes in host-seeking behavior due to ITBNs, IRS, and artificial light. It is important to determine whether these interventions impact the circadian clock itself or its output processes, and how they are being changed. Understanding this problem would contribute to the development of improved intervention methods to reduce the transmission of vector-borne diseases like malaria and arboviruses. Investigating the impact of selective pressure from ITBNs, IRS, and artificial light on mosquito populations and their circadian clocks is crucial for effectively combating vector-borne diseases. Further research in this field will provide insights into the underlying mechanisms and facilitate the development of interventions to alleviate the burden of these diseases on human populations.

**Section: Circadian interactions with “other clocks”**

Christopher Chabot

While much has been learned about the molecular mechanisms and locations of the circadian clocks that control overall behavior and physiology, much less is known about “other clocks” that allow for anticipation and synchronization to periodic environmental effects with different fundamental frequencies including tidal, lunar, and annual periodicities. Although the existence of endogenous clock systems that help organisms to synchronize to these periodic environmental changes has been demonstrated in many species [102], little is known about the location or underlying mechanisms of the clocks that might control them [103,104]. Perhaps most is known about the clocks that control circatidal rhythms and it appears that the clock system that controls these rhythms appear to be controlled by two phase locked “circadian” clocks (better termed “circalunidian”) each with a fundamental period of 24.8 hours yielding two bouts of activity phase locked at 180 degrees and producing behavioral rhythms with a period of 12.4 hours [102,105]. This led to speculation that these clocks may actually be assembled from the canonical circadian clock genes and, while early results focusing on *per* and *clk* did not support this hypothesis [106], a recent study in a marine amphipod suggests the involvement of a core circadian clock gene, *bmal1*, in the mechanism of the clocks controlling circatidal rhythms [107]. These results suggest that these clocks are also controlled by circadian clock-type transcription-translation negative feedback loops. In addition, protein kinases appear to be involved in the mechanisms underlying both circadian and circatidal rhythms [106]. As for circalunar rhythms, while these have been identified in a wide variety of marine organisms [108], even less is known about the molecular components of these clocks. While a few genetic “fishing expeditions” looking for rhythmic transcripts have proposed some possible targets for circalunar clocks [109], convincing data are not yet apparent and it does not appear that canonical circadian genes are part of these clocks. Likewise, while circannual rhythms have been identified in birds [110] and mammals [111], their molecular mechanisms and physiological control are completely unknown. Thus, there is much work to do in order to unravel these clocks and even more work to be done to understand the integration of these circadian, circatidal, and circalunar rhythms in the animals that possess them. A greater understanding of these diverse clock systems may enable us to better understand the origins and evolution of circadian clocks as well. In conclusion, further research is needed to unravel the molecular mechanisms and locations of the diverse clock systems that control periodic environmental effects with different fundamental frequencies. This research may have a significant impact on our understanding of the evolution of circadian clocks and may lead to new strategies for conditions related to disrupted circadian rhythms.

**Section: Allelic clock gene variation and species movements**

Seth J. Davis

Seasonal migration is a fascinating phenomenon observed in various plants and animals, including birds and fish. The migration patterns of these species are regulated by their internal circadian clocks, which are influenced by external environmental cues such as light and temperature. Understanding the molecular and genetic mechanisms underlying seasonal migration is an important area of research that could have implications for conservation efforts, as well as our understanding of the ecological and evolutionary factors driving migration. Researchers in this field may employ a variety of techniques, such as genetic sequencing and manipulation, physiological measurements, and tracking technologies to gain insights into the complex interactions between an organism’s circadian clock and its environment during seasonal migration. Further research in this area will deepen our understanding of the intricate biological processes that enable these remarkable migrations to occur, and inform our efforts to conserve and protect these species in a rapidly changing world. Several areas can be envisaged not that also are likely to be explored. For one, whole genome sequencing, phylogenies and haplotype analyses are powerful tools to understand the purpose of clock variation. These approaches provide a way to examine the evolution and diversification of clock genes across different species, allowing researchers to gain insights into how the clock has adapted to different environmental conditions and ecological niches. Comparative genomics can also identify clock genes that are under strong positive selection, providing clues about the adaptive significance of specific clock mechanisms. Furthermore, haplotype analyses can reveal the distribution of clock variants within and between populations, enabling researchers to investigate how natural selection and other factors have shaped clock variation in different contexts. Ultimately, these approaches can shed light on the functional consequences of clock variation, including effects on development, physiology, behavior, and fitness, and help us understand the role of circadian clocks in the broader context of evolutionary biology. The evolutionary history of circadian clock genes has been an active area of research for many years and will undoubtedly be critical future research. Researchers have used phylogenetic analyses to investigate the origins and diversification of clock genes across different kingdoms of life. The phylogenetic analyses of clock genes suggest that they originated early in the evolution of life, perhaps as far back as the last universal common ancestor. Furthermore, different kingdoms of life have independently evolved their own sets of clock genes, although there are some similarities in the structure and function of these genes. Recent studies have also begun to investigate the role of gene duplications and gene loss events in the evolution of clock genes. Overall, a better understanding of the evolutionary history of circadian clock genes can provide insights into the selective pressures that have shaped their function and diversity across different organisms.

**Section: New Imaging**

Seth J. Davis

The circadian system was unraveled using various imaging approaches. The utility of luciferase provided a powerful system to explore physiological questions and interrogate the underlying genetics via mutational and natural variation methods. Current mechanistic questions will examine the coupling of cell-cell communication. This will require the development of new fluorescent and other single-cell approaches to monitoring small molecules, nucleic acids and proteins that contribute to rhythmic processes. Similarly, it will be critical to have standardized approaches to multi-color imaging of two luminescent reporters to examine directly, and non-correlatively, how gene expression system provides a controlling node for another. In that, the utility of novel approaches of split fluorescent proteins and split luciferases will create an exiting new field on understanding when two proteins interact, when this interaction is in a certain cellular compartment, and when protein-protein interactions lead to changes in biochemistry and changes in gene expression of targets. Understanding the heterodimer relationship of transcription factor targets, compared to “known” understandings of the homodimers that are reported now deciphers the mechanistic details by which 10%–50% (or more) of a transcriptome can be rhythmic in a cell, tissue or organism. The impact of this research will be significant. A better understanding of the coupling mechanisms involved in circadian rhythms could lead to new mechanistic understandings of how rhythmic transcription in initiated by heterodimers. How hundreds of such factors coordinate tens of thousands of genes in tens to hundreds of tissues is a massive deconvolution problem. In this, imaging tools will be key to understand autonomous and non-autonomous relations of cells as they internally couple to generate organismal partners.

## Conclusion

In conclusion, the study of circadian rhythms is a rapidly evolving and multifaceted field with many unanswered questions. Recent advancements in imaging techniques, mathematical modeling, and genetic research have enabled scientists to gain a deeper understanding of the mechanisms that drive circadian rhythms. However, there is still much to learn, particularly regarding other clocks that allow organisms to synchronize with different periodic environmental effects. Over the next five to ten years, experts predict a continuation of research in these areas, as well as an increased focus on the role of whole genomes, phylogenies, and haplotype efforts to understand the purpose of clock variation. By gaining a better understanding of the evolutionary ontology of circadian clock genes, researchers hope to unlock further insights into the underlying genetic and physiological mechanisms of circadian rhythms. Overall, continued research in this field has the potential to not only advance our understanding of biological timekeeping but also yield important applications in medicine, agriculture, and beyond.
